# Automated shape-independent assessment of the spatial distribution of proton density fat fraction in vertebral bone marrow^☆^

**DOI:** 10.1016/j.zemedi.2022.12.004

**Published:** 2023-01-31

**Authors:** Tobias Haueise, Norbert Stefan, Tim J. Schulz, Fritz Schick, Andreas L. Birkenfeld, Jürgen Machann

**Affiliations:** a Section on Experimental Radiology, Department of Diagnostic and Interventional Radiology, University Hospital Tübingen, Tübingen, Germany; b Institute for Diabetes Research and Metabolic Diseases, Helmholtz Munich at the University of Tübingen, Tübingen, Germany; c German Center for Diabetes Research (DZD), Tübingen, Germany; d Department of Diabetology, Endocrinology and Nephrology, University Hospital Tübingen, Tübingen, Germany; e Department of Adipocyte Development and Nutrition, German Institute of Human Nutrition Potsdam-Rehbrücke, Nuthetal, Germany; f Institute of Nutritional Science, University of Potsdam, Potsdam, Germany

**Keywords:** Bone marrow, CSE MRI, Deep learning, PDFF, Segmentation

## Abstract

This work proposes a method for automatic standardized assessment of bone marrow volume and spatial distribution of the proton density fat fraction (PDFF) in vertebral bodies. Intra- and interindividual variability in size and shape of vertebral bodies is a challenge for comparable interindividual evaluation and monitoring of changes in the composition and distribution of bone marrow due to aging and/or intervention. Based on deep learning image segmentation, bone marrow PDFF of single vertebral bodies is mapped to a cylindrical template and corrected for the inclination with respect to the horizontal plane. The proposed technique was applied and tested in a cohort of 60 healthy (30 males, 30 females) individuals. Obtained bone marrow volumes and mean PDFF values are comparable to former manual and (semi-)automatic approaches. Moreover, the proposed method allows shape-independent characterization of the spatial PDFF distribution inside vertebral bodies.

## 1 Introduction

The composition and regional distribution of hematopoietic (red) bone marrow (BM) in the vertebral bodies is variable and subject to age- and gender-dependent changes in healthy individuals (healthy aging) [1], [2], [3], [4]. Furthermore, significant changes in composition can occur due to metabolic diseases (e.g. diabetes type 2), osteoporosis or hematological diseases [5], [6], [7], [8], [9]. In addition, there are various therapeutic strategies such as systemic chemotherapy and local radiotherapy that change the composition of BM [10], [11], [12], [13].

In order to study the properties of BM and to monitor changes without radiation exposure, quantitative magnetic resonance imaging (qMRI) and magnetic resonance spectroscopy (MRS) have been applied [14], [15], [16], [17]. In contrast to single voxel ^1^H-MRS, which has been considered the reference standard for the analysis of composition of BM but is limited to localized focal regions of a few cm^3^ and therefore not able to assess the spatial distribution of fat and water inside vertebral bodies, quantitative imaging techniques can be used to measure the bone marrow proton density fat fraction (BM_PDFF_) in a three-dimensional volume covering the complete anatomical region of interest. As vertebral bone marrow is characterized by short T2* times due to the presence of iron containing paramagnetic hematopoietic cells [18] and trabecular bone [17], multi-echo Dixon techniques with consideration of the spectral fat signal components and correction of the signal decay of the measured components are currently considered the gold standard for volumetric determination of BM_PDFF_ (e.g. 6-point Dixon [19]).

Quantitative assessment of BM_PDFF_ can be performed using different strategies: Evaluation of regions of interest (ROIs) (e.g. central circular ROIs [1], [5]), manual segmentation of the bone marrow cavity [20], [21], or automatic segmentation using deep learning incorporating multiple sagittal image slices [22]. However, all those approaches do not adequately consider the different size and interindividual variability in shape of the vertebral bodies: the regional distribution and inhomogeneity of BM_PDFF_ inside the vertebral body are often neglected. A standardized, shape-independent method which is able to monitor features of PDFF distribution inside vertebral bodies and to compare changes in subgroups regarding anthropometric and clinical parameters (e.g. insulin sensitivity) is still missing.

In this work, a shape-independent method for the standardized analysis of BM_PDFF_ inside vertebral bodies is presented. Therefore, a three-dimensional deep learning (DL)-based vertebral body segmentation model is used to generate shape-independent BM_PDFF_ distribution maps of the lumbar spine that allow, besides the quantification of BM_PDFF_, an interindividual analysis of sub-regional distribution patterns inside the vertebral bodies.

## 2 Materials and methods

### 2.1 Subjects

For testing the proposed method, imaging data of a study cohort of 60 healthy Caucasian volunteers without bone malignancies in their medical history (30 females) was analyzed. The subjects were split in two age groups for males and females using the median age of 50 years as threshold (15 females <50 years, 33.9 ± 9.6 years, 24.9 ± 7.3 kg/m^2^; 15 females >50 years, 59.9 ± 6.2 years, 26.1 ± 6.3 kg/m^2^; 15 males <50 years, 32.6 ± 8.3 years, 26.3 ± 4.9 kg/m^2^; 15 males >50 years, 60.0 ± 6.5 years, 26.9 ± 3.7 kg/m^2^). Differences in BMI between age groups were not significant for both genders (p = 0.4067 and p = 0.3837, respectively).

The study was approved by the local ethics committee and written informed consent was obtained from all subjects prior to participation.

### 2.2 MR examinations

MR examinations were performed on a 3 T whole-body scanner (Magnetom Vida, Siemens Healthcare, Erlangen, Germany). Subjects were positioned head first in supine position with a spine-array coil mounted on the patient table of the scanner. For homogeneous coverage of the body trunk, an 18-channel body-array coil was placed on the lower abdomen. A 3D volumetric interpolated breath-hold examination (VIBE) 6-point chemical-shift-encoding (CSE) Dixon sequence covering the lumbar spine was executed using the following parameters: matrix size 160x104, field-of-view 380x313 mm, in-plane voxel size 1.2 x 1.2 mm, section thickness 3 mm, TE = 1.09, 2.46, 3.69, 4.92, 6.15 and 7.38 ms, TR = 13 ms, flip angle 4°, encoding acceleration with Caipirinha, factor 2 in both, phase-encoding and slice encoding directions, bandwidth 1078 Hz/pixel, acquisition time TA = 17 s (breath-hold). The PDFF maps were generated inline on the console of the scanner applying the vendor’s algorithm, correcting for microscopic magnetic field inhomogeneities (T2*) and applying a multi-peak fat model [23].

For acquisition of training data for the DL model, a similar T1-weighted VIBE two-point Dixon sequence with 1.4 x 1.4 mm in-plane voxel size, 3 mm section thickness, TE = 1.23 ms and TR = 4.36 ms was applied.

### 2.3 Automated evaluation of BM_PDFF_ using deep learning-based image segmentation

For automated segmentation of the vertebral bodies, a DL-based 3D U-Net model (nnU-Net, full resolution configuration) [24] was derived from an independent stratified (gender, age, BMI) MRI data set of 30 subjects from local ongoing studies as described above (see Sec. 2.2). The manual labeling process of lumbar vertebrae was performed by a doctoral student (T.H.) under the supervision of two experienced medical physicists (>25 years of experience, F.S., J.M.).

The model was trained out-of-the-box (i.e. without the manual tuning of any hyperparameters) using a five-fold cross-validation scheme over 1000 epochs, providing the mean (i.e. the output of an ensemble) of the five independent models as resulting segmentation as suggested in [24].

The model-generated segmentations were evaluated using metrics of the class-wise confusion matrix as well as by the volumetric error (spatial accuracy of the model) compared to the manual ground truth segmentation from the training set as well as on a manually segmented stratified random sample of 12 subjects from the study cohort.

For further validation of the obtained BM_PDFF_ quantification from the trained DL model, an independent, open access reference database of lumbar BM_PDFF_ values (MyoSegmenTUM spine) [25] was used.

After application of the trained DL model on the four groups of the study cohort, volumetric characterization (mean, min, max and SD of all voxels) of BM_PDFF_ was derived from the automatic segmentations of the 6-point Dixon MR images by averaging over all voxels of the segmented vertebral body.

### 2.4 Creation of standardized BM_PDFF_ distribution maps

**Figure 1:**
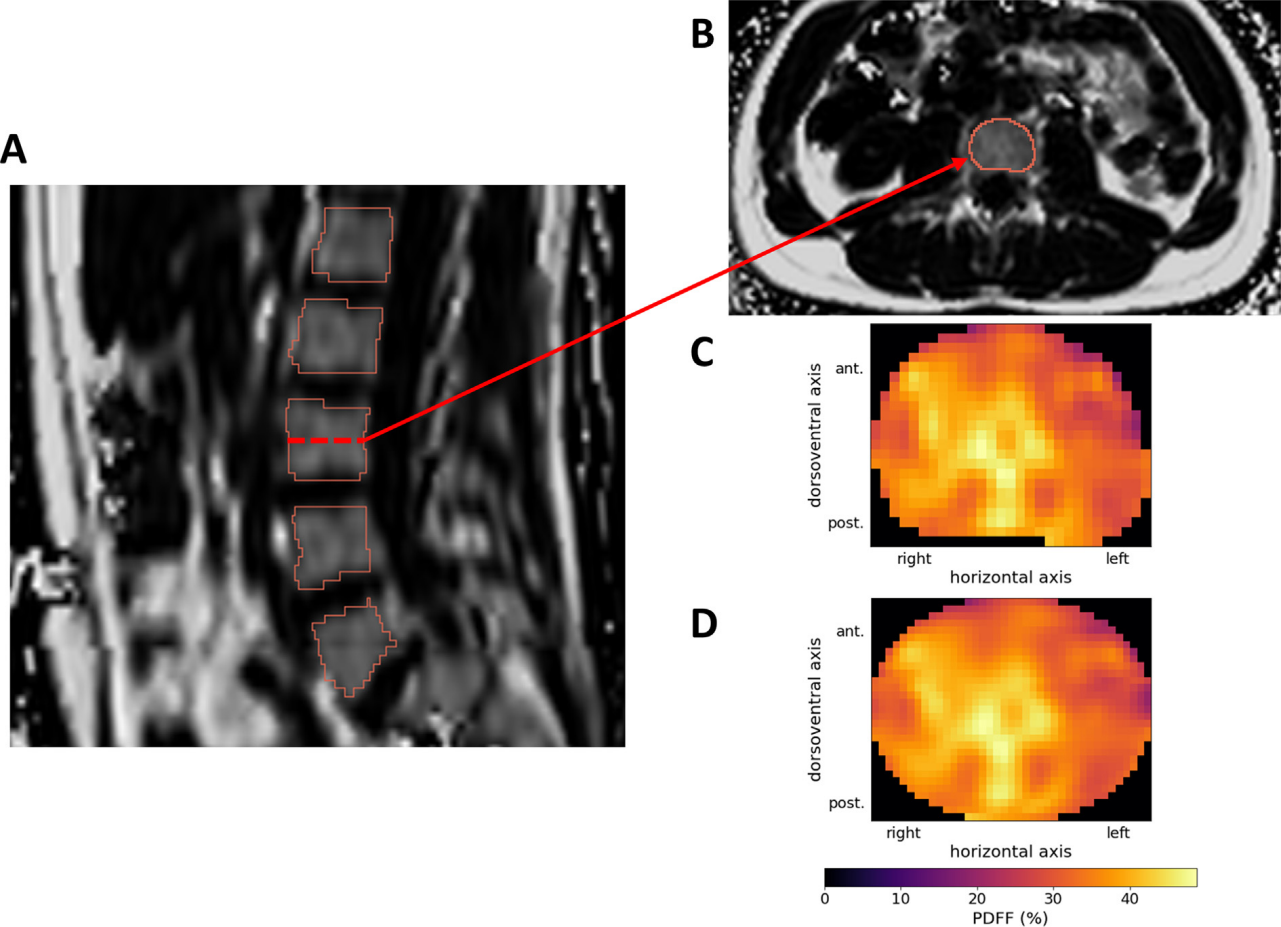
Workflow of the creation of standardized BM_PDFF_ distribution maps. Based on automated segmentation of vertebrae (**A**, **B**), raw BM_PDFF_ values (**C**) are mapped to a standardized cylindrical shape using linear interpolation along horizonal and dorsoventral axes (**D**).

Based on the automatically obtained segmentations (see Fig. 1A–C), each vertebral body is projected onto a standardized cylindrical shape using slice-wise linear interpolation of the PDFF map. After this registration of each vertebra to a standard cylindrical shape, three perpendicular BM_PDFF_ standardized distribution maps corresponding to the mid-transverse, mid-sagittal and mid-coronal plane of each vertebra can be derived and used for further analysis (see Fig. 1D).

The cylindrical shape is defined in terms of sampling points used for interpolation. In the mid-transverse plane of each vertebra, an elliptical base area is constructed using 20 and 16 sampling points on the major and minor semi-axes, respectively. First, the PDFF map is interpolated row-wise along the direction of the horizontal axis. Second, column-wise interpolation in the direction of the dorsoventral axis is performed. According to the elliptical template shape, the rows are resampled to yield the PDFF distribution map. The mid-coronal and mid-sagittal planes are constructed using the center-of-mass of the segmented vertebral body. Analogously, row-wise interpolation along the horizontal or dorsoventral axis is followed by interpolation in head-feet direction. Along the longitudinal axis, 20 sampling points are used.

To correct for the vertebral body inclination in the transverse plane, the segmented 3D PDFF image volume is rotated by detecting the inferior edge in the mid-sagittal plane of each vertebra. Its inclination angle is obtained using Hough transform of the segmentation map.

The extent to which the inclination correction has an influence is investigated by analyzing mean BM_PDFF_, range and SD of BM_PDFF_. SD is considered a measure of inhomogeneity of BM_PDFF_ inside the vertebral body.

The generated standardized distribution maps are compared to the corresponding raw PDFF image slices and the volumetric assessment from the segmentation model in terms of the metrics mentioned above.

### 2.5 Statistical tests

All data are reported as mean ± SD unless stated otherwise.

Performance of the segmentation model is evaluated using class-wise metrics of the confusion matrix, e.g. Dice similarity coefficient (DSC).

Bland-Altman plots are used to visualize the agreement between the automated volumetric quantification of BM_PDFF_ and the values derived from the proposed standardized distribution maps.

Welch’s t-test or Mann-Whitney u-test are selected to compare the different strategies for quantification and assessment of differences in anthropometric measures, as appropriate. *p*-values <0.05 were considered statistically significant.

All statistical analyses were performed in Python 3.8 using SciPy 1.8.0.

## 3 Results

### 3.1 DL-based segmentation

The evaluation of the cross-validated training metrics shows that the segmentation model precisely segments the vertebral bodies of the lumbar spine, both in terms of mean DSC (0.894 ± 0.026) and mean volumetric error (1.41 ± 0.26 ml, corresponding to 8.63 ± 2.15 %) across all lumbar vertebrae. The cross-validation metrics are summarized in Table 1.

**Table 1:** Mean validation metrics of the class-wise confusion matrix and standard deviation of the five-fold cross-validated model training using a total of 30 annotated data sets.

	L5	L4	L3	L2	L1
Accuracy	0.999 ± 0.001	0.999 ± 0.001	0.999 ± 0.001	0.999 ± 0.001	0.999 ± 0.001
DSC	0.848 ± 0.190	0.916 ± 0.087	0.907 ± 0.076	0.898 ± 0.106	0.900 ± 0.080
FDR	0.148 ± 0.193	0.077 ± 0.103	0.100 ± 0.098	0.106 ± 0.125	0.103 ± 0.103
FNR	0.132 ± 0.121	0.087 ± 0.084	0.081 ± 0.079	0.092 ± 0.106	0.088 ± 0.010
FOR	0.001 ± 0.001	0.001 ± 0.001	0.001 ± 0.001	0.001 ± 0.001	0.001 ± 0.001
FPR	0.001 ± 0.001	0.001 ± 0.001	0.001 ± 0.001	0.001 ± 0.001	0.001 ± 0.001
Jaccard	0.767 ± 0.200	0.856 ± 0.133	0.837 ± 0.118	0.828 ± 0.144	0.826 ± 0.113
NPV	0.999 ± 0.001	0.999 ± 0.001	0.999 ± 0.001	0.999 ± 0.001	0.999 ± 0.001
Precision	0.852 ± 0.193	0.923 ± 0.103	0.900 ± 0.098	0.894 ± 0.125	0.897 ± 0.103
Recall	0.868 ± 0.122	0.913 ± 0.084	0.919 ± 0.079	0.908 ± 0.106	0.912 ± 0.010
TNR	0.999 ± 0.001	0.999 ± 0.001	0.999 ± 0.001	0.999 ± 0.001	0.999 ± 0.001
Rel. error (%)	7.81 ± 7.62	6.07 ± 8.14	8.42 ± 7.27	8.85 ± 7.14	11.9 ± 7.79
Abs. error (ml)	1.65 ± 2.97	1.02 ± 1.31	1.33 ± 1.19	1.40 ± 1.18	1.65 ± 1.08

*FDR* false discovery rate, *FNR* false negative rate, *FOR* false omission rate, *FPR* false positive rate, *NPV* negative predictive value, *TNR* true negative rate.

On a random sample of the study cohort (n = 12, see Sec. 2.3), mean DSC (0.901 ± 0.024) and mean volumetric error (1.52 ± 0.29 ml, corresponding to 7.48 ± 1.24 %) of all vertebrae of the lumbar spine were found comparable within the standard deviation.

External validation using MyoSegmenTUM spine reference data [25] shows good agreement in terms of BM_PDFF_ values. For female and male subjects <50 years, differences in sample size and anthropometrics are not significant (15 females <50 years, 29.9 ± 7.1 years, 26.0 ± 1.6 kg/m^2^, p = 0.2039 and p = 0.5771, respectively; 15 males <50 years, 30.5 ± 4.9 years, 27.4 ± 2.8 kg/m^2^, p = 0.4117 and p = 0.4901, respectively). In both groups, differences in BM_PDFF_ of the lumbar spine are also not significant from L5–L1 compared to the study cohort (p = 0.6840–0.8525 for females and p = 0.1031–0.8176 for males, respectively).

### 3.2 Standardized distribution maps

The correction for the inclination of the vertebral body did not result in any significant change of the obtained mean, range or SD of BM_PDFF_ compared to their initial value without the correction in both the generated distribution maps as well as in the corresponding PDFF image slices. One exception was found for L5 in the group of females >50 years (p = 0.0357 and p = 0.0293 for the generated maps and the PDFF image slices, respectively).

Mean BM_PDFF_ obtained from generated distribution maps do not show any significant differences across genders and age groups compared to the values obtained from the PDFF image slices and the volumetric assessment.

A significant overestimation of the minimum BM_PDFF_ (up to 6.1 % for females >50 years compared to the mean volumetric BM_PDFF_) occurred in L1 for both genders and age groups (p = 0.0141 for females <50 years, p = 0.0017 for males <50 years, p < 0.001 for females >50 years, p = 0.0032 for males >50 years). For females in both age groups, a similar overestimation (up to 6.3 % for females >50 years) of the minimum BM_PDFF_ was found in L2. Correcting for the inclination angle reduces the difference to the volumetric minimum from 5.3 % for females >50 years up to non-significance in L5 for female subjects in both age groups (p = 0.1385 for females <50 years and p = 0.1664 for females >50 years, respectively).

The inhomogeneity of BM_PDFF_ as expressed by SD was significantly underestimated in L1 for all groups except females <50 years. Correcting for the inclination results in an approximation to the volumetric assessment but differences remain significant.

In some other cases (underestimation of SD in L2 and L3 for males >50 years, overestimation of the minimum in L4 for males <50 years), significant deviations from the corresponding volumetric assessment were found. By correction for the inclination, significant underestimations of SD in L4 for males of both age groups (p = 0.0817 for males <50 years, p = 0.0623 for males >50 years) in L5 for males <50 years (p = 0.4853) and of the maximum in L2 for males >50 years (p = 0.0639) are eliminated.

Except for the minimum BM_PDFF_, all other metrics from the generated distribution maps are highly correlated (R^2^ = 0,6813 for SD in males <50 years up to R^2^ = 0,9812 for mean BM_PDFF_ in females <50 years) with the volumetric BM_PDFF_. In most cases (except SD for females <50 years, maximum for females >50 years, mean and maximum for males >50 years) the inclination correction leads to a slightly worsened correlation between the values obtained from the generated distribution maps and the respective volumetric assessment.

**Figure 2:**
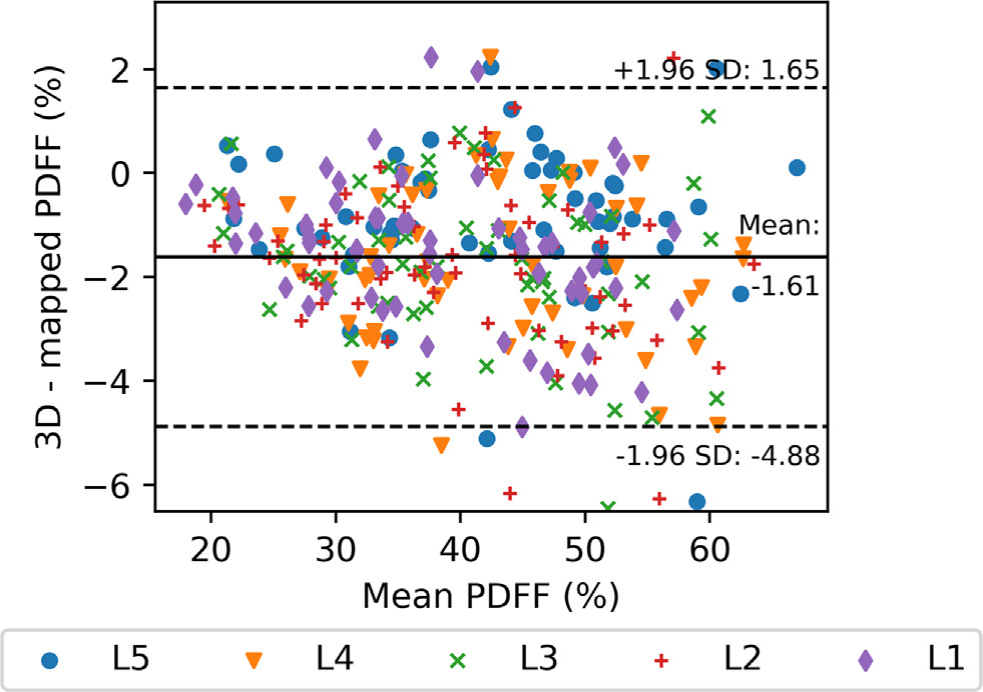
Bland-Altman plot showing agreement between BM_PDFF_ values derived from using all voxels from 3D segmentation and mean value of standardized distribution maps.

Regarding the mean BM_PDFF_, Bland-Altman analysis reveals a low negative bias of the generated distribution maps compared to the volumetric assessment (see Fig. 2).

**Figure 3:**
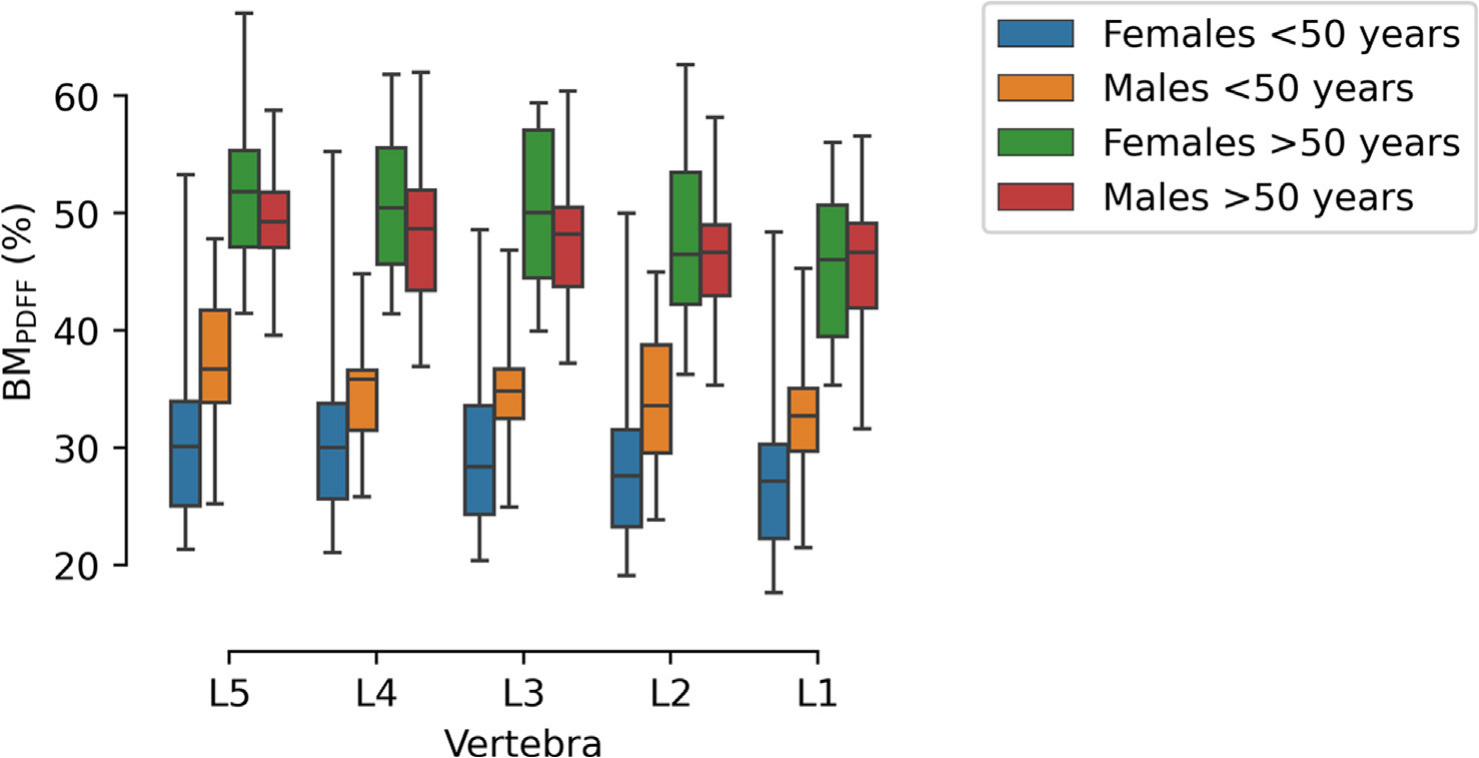
Quantified BM_PDFF_ of the lumbar spine in the four groups of the study cohort: females <50 years (blue), males <50 years (orange), females >50 years (green), males >50 years (red).

When applied to the study cohort, significant differences in mean BM_PDFF_ with respect to age are found in both genders and all vertebrae. Across genders, females and males <50 years show significant differences in mean BM_PDFF_ in all vertebrae except L4. Differences between females and males >50 years are not significant. Along the lumbar spine, mean BM_PDFF_ monotonically decreases from L5 to L1 (except in the group of females <50 years) (see Fig. 3).

The range of BM_PDFF_ extends from 35 % in L1 of females <50 years to 72 % in L5 of females >50 years. In females of both age groups, the range and inhomogeneity decrease from L5 to L1, whereas in males of both age groups the maximum range is found in L3 (see Table 2).

**Table 2:** BM_PDFF_ (%) in dependence of gender and age as obtained from the distribution maps.

		L5	L4	L3	L2	L1
Females <50	Min	9.9 ± 3.8	12.4 ± 5.9	9.9 ± 5.1	9.0 ± 3.9	7.6 ± 3.3
	Max	50.5 ± 14.6	50.6 ± 15.8	50.2 ± 16.5	46.0 ± 14.5	42.7 ± 11.4
	SD	6.5 ± 2.5	5.8 ± 2.2	6.4 ± 2.4	5.6 ± 2.4	4.9 ± 1.8
Males <50	Min	12.1 ± 5.2	12.8 ± 5.0	7.3 ± 4.9	10.1 ± 5.2	10.4 ± 5.3
	Max	57.4 ± 6.8	54.5 ± 6.4	60.9 ± 7.3	60.9 ± 14.1	54.8 ± 10.4
	SD	7.5 ± 1.4	6.8 ± 1.3	8.2 ± 1.1	7.6 ± 2.9	5.9 ± 1.2
Females >50	Min	18.0 ± 5.2	18.4 ± 6.0	17.0 ± 5.8	15.9 ± 6.1	13.3 ± 5.3
	Max	89.2 ± 15.7	85.6 ± 15.6	80.8 ± 13.7	78.4 ± 13.5	73.9 ± 9.4
	SD	11.3 ± 3.1	10.6 ± 3.0	10.5 ± 2.3	10.0 ± 2.5	9.2 ± 1.6
Males >50	Min	18.5 ± 8.4	16.6 ± 8.4	10.6 ± 5.8	11.4 ± 6.7	11.8 ± 4.2
	Max	82.0 ± 14.6	79.1 ± 12.0	76.3 ± 13.8	76.1 ± 7.7	71.6 ± 10.0
	SD	8.8 ± 3.2	9.1 ± 1.5	9.8 ± 1.6	9.8 ± 1.8	8.6 ± 1.4

**Figure 4:**
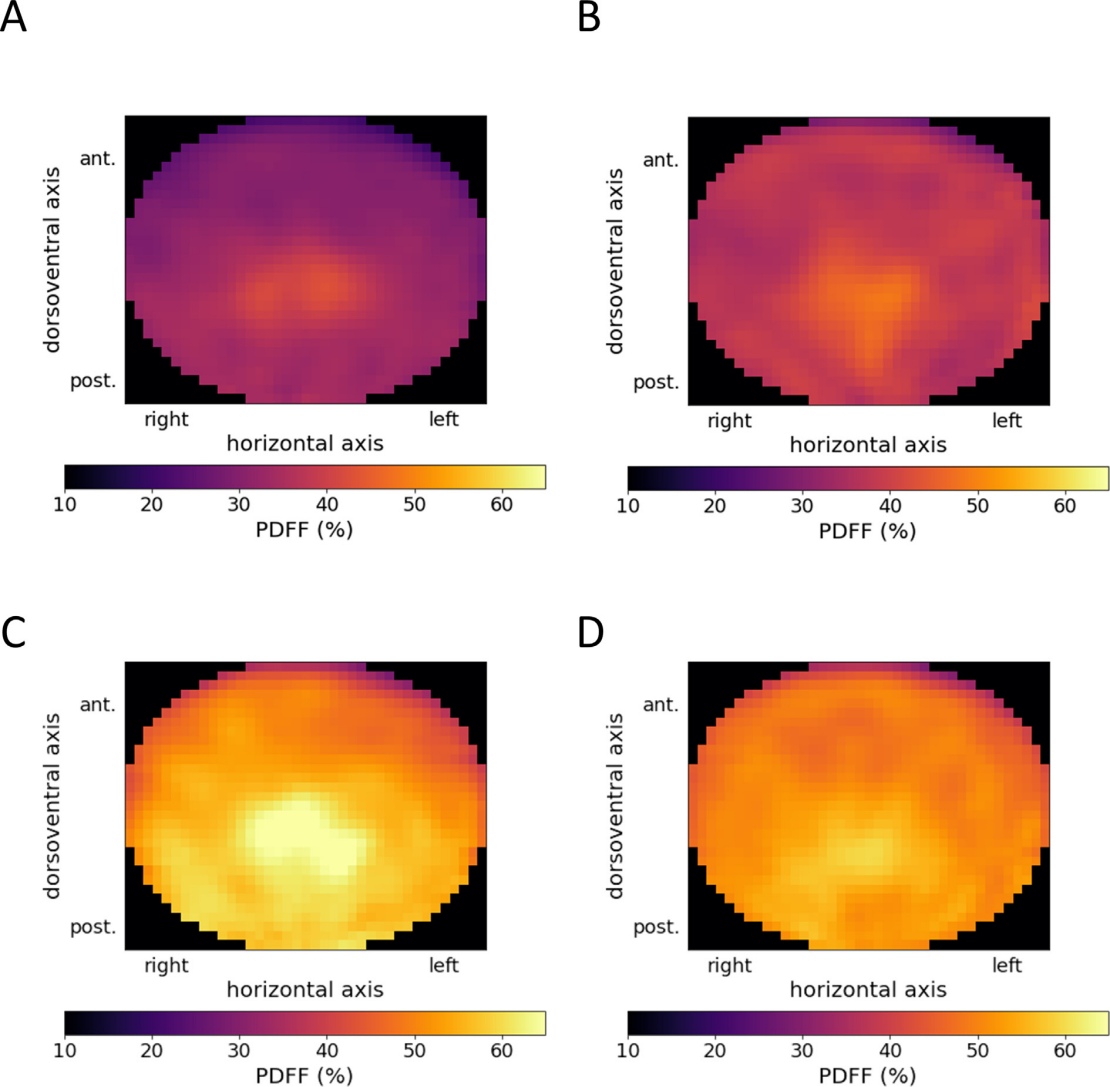
Standardized horizontal distribution maps of L3 showing qualitative differences between the four groups of the study cohort: females <50 years (**A**), males <50 years (**B**), females >50 years (**C**), males >50 years (**D**).

Qualitatively, the distribution maps show variable patterns across age groups and gender (see Fig. 4). The increase of mean BM_PDFF_ as well as the qualitative increase of inhomogeneity can be displayed for the purpose of visual inspection.

## 4 Discussion

Inhomogeneous composition and distribution of PDFF in vertebral (red) bone marrow (BM_PDFF_) as well as intra- and interindividual spatial variability between vertebral bodies along the spine and across different subjects pose a particular challenge on the systematic, reproducible and standardized evaluation of BM_PDFF_. In this work, the proposed method tackles these challenges by generating standardized distribution maps of vertebral bodies corrected for their inclination. These maps show high correlation with mean BM_PDFF_, its range and its quantitative inhomogeneity obtained from the direct unstandardized volumetric assessment using DL-based image segmentation. The presented segmentation model allows for the precise and fully automated (and therefore time-saving) quantification of BM_PDFF_ similar to strategies reported in the literature [20], [25], [26], [27]. The proposed standardized registration of vertebral bodies on a unified spatial structure allows both, direct shape-independent comparison of different vertebral bodies of an individual subject as well as interindividual comparability or analyses in anthropometrically matched groups. As a downside, the method relies on DL-based image segmentation as an expensive (in terms of time and computational complexity) preprocessing step that also needs a manually segmented training data set. Training data were labeled in fat-selective images but – due to the heterogenous composition of bone cavity – delineation will probably lead to identical results using other contrasts as water selective, in-phase and/or opposed phase images available from the applied CSE sequence. However, a 3D dataset with sufficient spatial resolution is mandatory in order to avoid partial volume effects by inclusion of structures assigned to compact bone. Reported under- or overestimations of single metrics (e. g. minimum BM_PDFF_) do not show a clear systematic, but are related to each other: overestimation of the minimum (as found in L1) decreases the range of possible values of BM_PDFF_ thereby reducing the standard deviation which is expressed in an underestimation of BM_PDFF_ inhomogeneity.

Spatial standardization to provide an interindividual comparison of MRI-assessed biomarkers is common practice in other body regions as CSE-based MRI enables reliable assessment and depiction of fat and, especially, its distribution in different organs. For example, the body structure is standardized for the assessment of whole-body adipose tissue topography along the longitudinal axis of the body [28]. When multi-point Dixon techniques are applied to organs such as the pancreas [29], [30] or skeletal muscle [31], [32] to quantify ectopic lipids, or for the quantification of intrahepatic lipids [23], [33], the distribution of fat has mainly been taken under consideration in the pancreas differentiating fat content for head, body and tail [34] or skeletal muscle [32]. On the other hand, the liver is mostly considered as a whole by selecting a dedicated ROI in a representative part of the tissue. Thus, both approaches are found in the evaluation of BM_PDFF_ [1], [2], [3], [17].

Given the high reproducibility of CSE-based MRI between platforms of different field strengths and vendors [35], [36], it furthermore enables detection of short-term [34] and long-term changes [37] in PDFF. Using the proposed method, the standardized analysis of short- and long-term changes might also be of interest for the clinical and epidemiological characterization of bone marrow, e.g. in the field of hematological diseases [12], [13], [38], osteoporosis [8], [39], [40], Modic change classification, i.e. pathological alterations by endplate degeneration with subchondral bone marrow changes [41] as well as after clinically indicated physical inactivity [42] or in analysis of interindividual and matched distribution patterns in large epidemiological studies like UK Biobank [43] or the German National Cohort [44].

### 4.1 Limitations

First, the relatively small number of subjects (n = 15, each with 5 vertebral bodies) in analyzed subgroups harms the generalizability of the results such that a potential broader variability including special cases may not be adequately covered. An epidemiological study studying the number and type of vertebral deformities [39] indicates that vertebral deformities occur mostly along the longitudinal axis (crush, wedge or biconcave deformities). Therefore, the proposed method based on the mid-transverse plane of the vertebrae should not be impaired. Furthermore, the study was not carried out to assess differences in age- and gender subgroups with high statistical significance, but to test a strategy for anatomically standardized assessment of BM_PDFF_ and its distribution in vertebral bodies. An extension to subjects with, e.g., osteoporosis is subject to future studies. Second, the study is based on the hypothesis that the deep learning-based automatic segmentation of vertebral bodies allow for an accurate quantification of BM_PDFF_. Although this assumption seems reasonable, further research may focus on its verification. Third, there are no data on metabolic status and/or on bone health available in the study cohort which might allow an evaluation of spatial BM_PDFF_ distribution in relation to metabolic alterations (as insulin resistance, type 2 diabetes) and/or osteoporosis. Forth, the application of the method is only shown on the vertebrae of the lumbar spine. However, the translation to images covering the whole spine is possible.

## 5 Conclusion

The proposed method based on automated deep learning-based image segmentation can not only be used for robust and precise measurement of BM_PDFF_ based on the segmentation of the whole vertebral body but also to visualize its spatial distribution pattern inside the vertebral body. The method can serve as a tool to overcome biases in the assessment of representative BM_PDFF_ originating from varying quantification techniques and enables the systematic analysis of distribution patterns in groups of individuals. Further investigations of these distributions have the potential to deepen the understanding of the functionality of vertebral BM_PDFF_ and may thus help in evaluation of large-scale epidemiological studies as well as in follow-up analyses in patients with hematological diseases or therapy response.

## Declaration of Competing Interest

The authors declare that they have no known competing financial interests or personal relationships that could have appeared to influence the work reported in this paper.
